# HIV-1 protease inhibitor drug resistance in Kenyan antiretroviral treatment-naive and -experienced injection drug users and non-drug users

**DOI:** 10.1186/s12981-015-0070-y

**Published:** 2015-08-15

**Authors:** Valentine Budambula, Francis O. Musumba, Mark K. Webale, Titus M. Kahiga, Francisca Ongecha-Owuor, James N. Kiarie, George A. Sowayi, Aabid A. Ahmed, Collins Ouma, Tom Were

**Affiliations:** Department of Environment and Health Sciences, Technical University of Mombasa, Mombasa, Kenya; Department of Biomedical Science and Technology, Maseno University, Maseno, Kenya; Centre for Virus Research, Kenya Medical Research Institute, Nairobi, Kenya; Department of Pharmacy and Complementary Medicine, Kenyatta University, Nairobi, Kenya; Department of Medicine, Therapeutics, Dermatology and Psychiatry, Kenyatta University, Nairobi, Kenya; Department of Obstetrics and Gynecology, University of Nairobi, Nairobi, Kenya; Department of Medical Laboratory Sciences, Masinde Muliro University of Science and Technology, Kakamega, Kenya; Bomu Hospital, Mombasa, Kenya; Health Challenges and Systems Program, African Population and Health Research Centre, Nairobi, Kenya; Department of Medical Laboratory Sciences, Masinde Muliro University of Science and Technology, P. O. Box 190, Kakamega, 50100 Kenya

**Keywords:** HIV-1, Protease inhibitor, Drug resistance, Antiretroviral, Treatment-naive, Treatment-experienced, Injection drug users, Non-drug users, Coastal Kenya

## Abstract

**Background:**

Although injection drug use drives antiretroviral drug resistance, the prevalence of protease inhibitor (PI) resistance among Kenyan IDUs remains undetermined. We, therefore, explored PI resistance mutations and their association with viral load and CD4+ T cell counts in HIV-1 infected IDUs (ART-naive, n = 32; and -experienced, n = 47) and non-drug users (ART-naive, n = 21; and -experienced, n = 32) naive for PI treatment from coastal Kenya.

**Results:**

Only IDUs harboured major PI resistance mutations consisting of L90M, M46I and D30 N among 3 (6.4 %) ART-experienced and 1 (3.1 %) -naive individuals. Minor PI mutations including A71T, G48E, G48R, I13V, K20I, K20R, L10I, L10V, L33F, L63P, T74S, V11I, and V32L were detected among the ART-experienced (36.2 vs. 46.9 %) and -naive (43.8 vs. 66.7 %) IDUs and non-drug users, respectively. All the four IDUs possessing major mutations had high viral load while three presented with CD4+ T cell counts of <500 cells/ml. Among the ART-naive non-drug users, CD4+ T cell counts were significantly lower in carriers of minor mutations compared to non-carriers (*P* < 0.05).

**Conclusion:**

Transmitted drug resistance may occur in IDUs underscoring the need for genotyping resistance before initiating PI treatment.

## Background

Despite up-scaling of antiretroviral drugs for treatment and prevention, HIV-1 remains an important cause of infectious disease burden in the world. In the year 2013, an estimated 35 million people globally were infected with HIV of whom 24.7 million reside in Sub-Saharan Africa [1]. Substance consumption including injection drug use is an emerging epidemic that is driving the high HIV burden on the continent [2]. Among the 243 million people using substances in the world, 12.7 million are injection drug users (IDUs) of whom 13.1 % are living with HIV [1]. Africa is home to nearly 1.02 million IDUs of which 12.1 % are harbouring HIV infections [1]. In Kenya, about 18.3 % of the 49,000 IDUs, mainly concentrated in Nairobi and Coastal regions, are living with HIV [3]. Non-drug users are individuals not consuming illicit drugs classified by the United Nations Office on Drug and Crime (UNODC) [1]. However, non-drug users are also at increased risk of acquiring HIV infection, largely through engaging in unprotected and high risk sexual activities with IDUs [4–6]. As such, effective interventions are urgently required to reduce the high burden of HIV in these most-at-risk-populations.

The current standard regimen for first-line HIV treatment in Kenya consists of two nucleoside reverse transcriptase inhibitors (NRTI) mainly tenofovir disoproxil fumarate (TDF) or azidovudine (AZT), lamuvudine (3TC) and one non-nucleoside reverse transcriptase inhibitor (NNRTI) efevirenz (EFV) or nevirapine (NVP) [7]. Protease inhibitor (PI) drugs serve as HIV-1 pre- and post-exposure prophylaxis and substitute treatment in cases of first line antiretroviral treatment failures and/or intolerance [8]. Protease inhibitors are recommended in cases of virological failure, defined as a viral load >1000 copies/ml after 12 months of therapy or an increase in the viral load after initial viral suppression [7]. Consequently, PIs have been effective in further lowering HIV-associated morbidity and mortality [9, 10]. However, continued use of PIs as effective salvage treatment is hindered by emergence of drug resistance, largely due to poor accessibility and adherence to treatment [11]. Two classes of point mutations in the protease gene, designated major and minor mutations, mediate resistance to PIs [12]. Major mutations decrease susceptibility to PI drugs leading to poor virologic response and treatment failure, while minor mutations only enhance viral replication in the presence of major mutations [12]. In addition, previous studies reporting higher rates of major and minor PI resistance (24 vs. 8 %) among ART-experienced PI-naive IDUs compared to non-drug users [10], suggest spontaneous emergence of PI resistance in PI-naive individuals. To our knowledge, no study has concurrently determined rates of PI resistance in ART-naive and -experienced IDUs and non-drug users among PI treatment-naive individuals.

Several studies across Kenya show widespread resistance to NRTIs and non-NNRTIs in ART-naive and -experienced adults and children, breastfeeding infants and IDUs [13–31]. More importantly, previous studies showing 13.8 % resistance rates to first line (NRTIs and NNRTIs) ART regimens among IDUs from Mombasa, coastal Kenya, suggest existence of resistance to these drugs that are commonly used in combination with PIs [27]. In such cases of suspected drug resistance, the NNRTIs (NVP or EFV) or NRTIs (ABC) are often substituted with PIs (either LPV/r or ATV/r) as second-line treatment [7]. In addition, resistance to integrase inhibitors has also been detected in the country [32]. While a number of studies in the country show existence of resistance to PIs in ART-naive and -experienced individuals in adults and children from the general population, and treatment-naive female sex workers [16, 18, 29, 33, 34] no studies to date have established PI resistance in Kenyan IDUs. Since, high drug injection intensity particularly under the selection pressure of antiretroviral therapy accelerates emergence of resistance [35], this cross-sectional study, examined PI resistance in ART-naive and -experienced IDUs from Mombasa, a coastal city in Kenya.

## Methods

### Study design and population

This cross-sectional study was conducted in HIV-1 infected IDUs and non-drug using controls at Bomu Hospital, a social enterprise facility in Mombasa City, coastal Kenya. Injection drug users were recruited via respondent-driven, snowball and makeshift-outreach sampling methods. In the context of the current study, IDUs comprised of individuals exhibiting injection needle-stick scars and reporting a history of injecting any illicit drug classified by UNODC at least once in the previous month [1] while non-drug users were individuals reporting having never used any of the substances and drugs in the UNODC registry [1]. HIV-1 infection status of consenting IDUs and non-drug users was confirmed using Determine™ (Abbott Laboratories, Tokyo, Japan) and Unigold™ (Trinity Biotech Plc, Bray, Ireland) in accordance with the Kenya’s guidelines for adult HIV testing [36]. Those yielding positive results on both tests were subsequently recruited into the study. In addition, ART-naive study participants had not been initiated on ART. The ART-experienced study participants were on first-line ART (NRTIs and NNRTIs) consisting of TDF or AZT + 3TC + NVP or EFV [37]. None of the participants were on PIs.

### Clinical evaluations

In brief, about, 3.0 ml venous blood was collected from study participants in ethylenediaminetetraacetic acid vacutainer tubes (Becton–Dickinson, Franklin Lakes, USA) and used for enumerating CD4+ T cells, and plasma harvesting for HIV-1 viral load quantification. A drop of whole blood was applied on Whatman-FTA^®^ 903 cards (Schleicher & Schuell BioScience, Dassel, Germany) for preparing dry blood spots (DBS) that were stored at −20 °C until used for pro-viral DNA extraction. For enumerating CD4+ T cell counts, 5.0 μl of blood were stained with CD3/CD4/CD45 monoclonal antibody reagent (BD Tritest) and analysed on BD FACSCalibur™ according to the manufacturer’s protocols. HIV-1 viral load was determined in 0.2 ml plasma samples using the Abbott Molecular m2000sp sample preparation and m2000rt real-time amplification and detection systems (Abbott Molecular Inc., Illinois, USA) according to the manufacturer’s methods.

### HIV-1 DNA extraction and amplification

HIV-1 pro-viral DNA was extracted from DBS using QIAamp™ DNA blood mini kit (QIAGEN, Valencia, USA) according to the manufacturer’s recommendations. HIV-1 pro-viral DNA encoding protease gene was amplified by nested PCR with primers; Nyupol_7 (5′-GGGAATTTTCTTCAGAGCAG-3′) HXB2 nucleotide position 2125–2144, and Nyupol_8 (5′-TCTTCTGTCAATGGCCATTGT-3′) HXB2 nucleotide position 2635–2615 in the first round. Nyupol_9 (5′-TCCTTAACTTCCCTCAAATCACT-3′) HXB2 nucleotide position 2241–2264 and Nyupol_10 (5′-CTGGCACGGTTTCAATAG GACT-3′) HXB2 nucleotide position 2577–2556 were used in the second round. First and second round PCR was conducted in a 25 µl volume containing 5 µL DNA, 1× PCR buffer, 1.5 mM MgCl_2_, 1 U/µL* Taq* polymerase (KemTaq^®^), 200 µM of each dNTP (Invitrogen™, Carlsbad, USA), and 0.5 µM of each first or second round primers. Amplification consisted of initial denaturation at 94 °C for 4 min, followed by 40 cycles of 94 °C for 15 s, 50 °C for 30 s, and 72 °C for 1 min, with a final extension of 72 °C for 5 min. PCR products were electrophoresed in 1 % ethidium bromide-stained agarose gel, and visualized under ultraviolet light. PCR product sizes of approximately 330 bp were diagnostic for HIV-1 pro-viral DNA.

### HIV-1 protease inhibitor drug resistance mutations

PCR amplicons were purified with QIAquick™ PCR purification kit (Qiagen, Valencia, USA) according to the manufacturer’s protocol. Direct sequencing was performed using BigDye™ sequencing chemistry (Applied Biosystems^®^, Foster City, USA) on an ABI PRISM^®^ 3100 Genetic Analyzer (Applied Biosystems^®^, Foster City, USA) as per the manufacturer’s protocols. Primer pair Nyupol_9 and Nyupol_10 was used for forward and reverse sequencing reactions, respectively. Forward and reverse chromatograms were visually inspected and edited for base mis-calls using MEGA version 6.0 [38]. Pair-wise alignment and contiguous sequences were generated using DNA Baser Sequence Assembler version 4.20.0 (Heracle BioSoft, http://www.DnaBaser.com). Protease inhibitor drug resistance mutations were identified and interpreted using Stanford web-based HIV drug resistance database [39, 40] and the International AIDS Society-USA (IAS-USA) drug resistance panel [12].

### Data analysis

Data analysis was conducted using IBM^®^ SPSS 20.0 (IBM^®^ SPSS Statistics for Windows, Version 20.0. Armonk, NY: IBM Corp.). Age and CD4+ T cell counts and viral load were compared across study groups using Kruskal–Wallis test and Dunn’s post hoc correction for multiple comparisons. Differences in distribution of gender were compared across the study groups using Chi-square test. Rates of resistance mutations in each study group were summarised as numbers and proportions. Statistical significance was set at *P* < 0.05.

### Ethical considerations

This study was conducted following ethical approval obtained from Kenyatta University Ethical Review Committee, approval number PKU019/116 of 2012 in accordance with Helsinki declaration [41]. Written informed consent was obtained from all participants prior to enrolment into the study. Confidentiality was maintained throughout the study.

## Results

### Baseline characteristics of the study participants

Baseline characteristics of the study participants are presented in Table 1. Recruitment was within HIV-infected cohort thus sero-conversion rate is unknown. A total of 132 HIV-1 sero-positive study participants were successfully sequenced for protease inhibitor drug resistance. The study participants consisted of IDUs (ART-naive, n = 32 and -experienced, n = 47) and non-drug users (ART-naive, n = 21 and -experienced, n = 32). Gender distribution was similar among the study groups (*P* = 0.531). Age was significantly different across the groups (*P* = 0.009) with ART-naive IDUs (median, 30.1; IQR, 7.2) having younger individuals relative to ART-naive non-drug users (median, 36.0; IQR, 13.8; *P* < 0.05). In addition, 20 (42.5 %), 24 (51.1 %) and 3 (6.4 %) ART-experienced IDUs while 3 (9.3 %), 7 (21.9 %) and 22 (68.8 %) ART-experienced non-drug users were on antiretroviral therapy for <1, 1–3, and >3 years, respectively. The CD4+ T cell counts were similar across the study groups (*P* = 0.379). The HIV-1 viral load was significantly different across the study groups (*P* = 0.004) with ART-naive (median, 3.4; IQR, 2.7; *P* < 0.05) and -experienced (median, 3.5; IQR, 2.7; *P* < 0.01) IDUs presenting with lower levels compared to ART-naive non-drug users (median, 4.5; IQR, 2.8).

**Table 1:** Baseline characteristics of the study participants

Characteristic	Non-drug users		Injection drug users		*P*
	ART (−), *n* = 21	ART (+), *n* = 32	ART (−), *n* = 32	ART (+), *n* = 47	
Female, n (%)	16 (76.2)	20 (62.5)	18 (56.3)	30 (63.8)	0.531
Age, years	36.0 (13.8)	35.2 (10.6)	30.1 (7.2)^a^	31.6 (7.4)	*0.009*
Duration (years) on ART, n (%)					
<1	0 (0.0)	3 (9.3)	0 (0.0)	20 (42.5)	
1–3	0 (0.0)	7 (21.9)	0 (0.0)	24 (51.1)	–
>3	0 (0.0)	22 (68.8)	0 (0.0)	3 (6.4)	
CD4+ T cell counts/ml	568 (673)	412 (554)	532 (472)	426 (423)	0.379
Log_10_ HIV-1 RNA, copies/ml	4.5 (2.8)	4.3 (1.6)	3.4 (2.7)^a^	3.5 (2.7)^b^	*0.004*

Data are presented as medians (IQR, interquartile range) or indicated as number (n) and proportion (%) of subjects

*ART (−)* anti-retroviral treatment-naive, *ART (+)* antiretroviral treatment-experienced, *HIV-1* human immunodeficiency virus-1

^a^ *P* < 0.05 vs. ART (−) non-drug users

^b^ *P* < 0.01 vs. ART (−) non-drug users

Significant *P* values are shown in italic

### Protease inhibitor drug resistance

Major and minor HIV-1 protease drug resistance mutations detected in the study participants are shown in Table 2. Although major PI resistance mutations was not detected in the non-drug users, three major PI resistance mutations including L90M, M46I and D30N were detected in 4 (5.1 %) IDUs. Interestingly, mutation L90M co-existed with K20R minor mutation in 1 (2.1 %) ART-experienced IDU, while D30N co-existed with T74S+K20R minor mutations in 1 (3.1 %) ART-naive IDU. In addition, D30N+M46I co-existed with G48E and K20I minor mutations, respectively, in 2 (4.2 %) ART-experienced IDUs. Fourteen (29.8 %) ART-experienced IDUs harboured only minor mutations comprising of 1(2.1 %) K20I, 7 (8.5 %) K20R, 3 (6.4 %) L10I, 1 (2.1 %) V32L and 2 (12.5 %) L10V+K20R. In addition, 13 (40.6 %) ART-naive IDUs had only minor mutations including 1 (3.1 %) G48E, 1 (3.1 %) G48R, 5 (15.6 %) K20R, 2 (6.3 %) L10I, 1 (3.1 %) L10V, 1 (3.1 %) L101+K20R and 2 (6.3 %) L10V + K20R.

**Table 2:** Protease inhibitor drug resistance mutations

Mutation	Non-drug users		Injection drug users	
	ART (−), *n* = 21	ART (+), *n* = 32	ART (−), *n* = 32	ART (+), *n* = 47
**L**90**M** + K20R	0 (0.0)	0 (0.0)	0 (0.0)	1 (2.1)
**D**30**N** + T74S + K20R	0 (0.0)	0 (0.0)	1 (3.1)	0 (0.0)
**D**30**N** + **M**46**I** + G48E	0 (0.0)	0 (0.0)	0 (0.0)	1 (2.1)
**D**30**N** + **M**46**I** + K20I	0 (0.0)	0 (0.0)	0 (0.0)	1 (2.1)
G48E	0 (0.0)	0 (0.0)	1 (3.1)	0 (0.0)
G48R	0 (0.0)	0 (0.0)	1 (3.1)	0 (0.0)
K20I	1 (7.1)	0 (0.0)	0 (0.0)	1 (2.1)
K20R	8 (38.1)	5 (15.6)	5 (15.6)	7 (8.5)
L10I	2 (9.5)	0 (0.0)	2 (6.3)	3 (6.4)
L10V	0 (0.0)	2 (6.3)	1 (3.1)	0 (0.0)
L33F	0 (0.0)	2 (6.3)	0 (0.0)	0 (0.0)
V32L	0 (0.0)	0 (0.0)	0 (0.0)	1 (2.1)
I13V + L63P	0 (0.0)	1 (3.1)	0 (0.0)	0 (0.0)
L10I + K20R	1 (7.1)	0 (0.0)	1 (3.1)	0 (0.0)
L10V + K20R	0 (0.0)	1 (3.1)	2 (6.3)	2 (4.3)
L10V + T74S	0 (0.0)	3 (9.4)	0 (0.0)	0 (0.0)
L10V + V11I	2 (9.5)	0 (0.0)	0 (0.0)	0 (0.0)
L33F + A71T	0 (0.0)	1 (3.1)	0 (0.0)	0 (0.0)

Data presented are number and proportion of subjects. Mutations are denoted based on Stanford drug resistance database and International Antiviral Society-USA drug resistance mutations panel [12, 39, 40], where number shows amino acid position in the protease gene, and letter before position is wild type amino acid and after the position mutant amino acid

Major mutations are shown in bold

*A* alanine, *D* aspartic acid, *E* glutamic acid, *F* phenylalanine, *G* glycine, *I* isoleucine, *K* lysine, *L* leucine, *M* methionine, *N* asparagine, *P* proline, *R* arginine, *S* serine, *T* threonine, *V* valine, *ART (−)* antiretroviral treatment-naive, *ART (+)* antiretroviral treatment-experienced, *HIV-1* human immunodeficiency virus type-1

In non-drug users, 15 (46.9 %) ART-experienced individuals had minor mutations consisting of 5 (15.6 %) K20R, 2 (6.3 %) L10V, 2 (6.3 %) L33F, 1 (3.1 %) I13V + L63P, 1 (3.1 %) L10V + K20R, 3 (9.4 %) L10V + T74S and 1 (3.1 %) L33F + A71T. In addition, 14 (66.7 %) ART-naive non-drug users harboured minor mutations comprising of 1 (7.1 %) K20I, 8 (38.1 %) K20R, 2 (9.5 %) L10I, 1 (7.1 %) L101 + K20R, and 2 (9.5 %) L10V + V11I.

### Association of protease inhibitor resistance with viral load and CD4+ T cell counts

Viral load and CD4+ T cell counts of individuals with major and minor mutations are presented in Tables 3 and 4, respectively. All the four individuals harbouring major mutations presented with high viral load of 4624, 291,124, 141,341 and 209,871 copies/ml, which was greater than 1000 copies/ml viral load threshold required for viral suppression in the community [42]. In addition, three of the individuals with major mutations presented with CD4+ T cell counts of 473, 39, and 162 cells/μl which were <500 cells/μl. Carriage of minor mutations was not associated with viral load in all the study groups (*P* > 0.05), but among ART-naive non-drug users, CD4+ T cell counts were significantly lower in carriers of minor mutations compared to non-carriers (median, 455; IQR , 599 vs. 989, 507; *P* = 0.005).

**Table 3:** HIV-1 viral load and CD4+ T cell counts of individuals with major protease inhibitor drug resistance mutations

ID	Mutation	Age, years	Gender	ART duration, years	HIV-1 RNA (log_10_ HIV-1 RNA), copies/ml	CD4+ T cells/ml
#1	**D**30**N** + **M**46**I** + G48E	36.4	M	<0.5	4624 (3.7)	473
#2	**L**90**M** + K20R	34.6	M	1–3	291,124 (5.5)	637
#3	**D**30**N** + **M**46**I** + K20I	30.1	F	1–3	141,341 (5.2)	39
#4	**D**30**N** + T74S + K20R	33.3	M	Naive	209,871 (5.3)	162

Mutations are denoted according to Stanford drug resistance database and International Antiviral Society-USA drug resistance mutations panel [12, 39, 40]

Major mutations are shown in bold

*D* aspartic acid, *E* glutamic acid, *G* glycine, *I* isoleucine, *K* lysine, *L* leucine, *M* methionine, *N* asparagine, *S* serine; *T* threonine, *ART* antiretroviral therapy, *HIV-1* human immunodeficiency virus type-1

**Table 4:** Association between minor protease inhibitor resistance mutations, viral load and CD4+ T cell count

	Injection drug users					
	ART (−)			ART (+)		
	Mutation (−), *n* = 18	Mutation (+), *n* = 13	*P*	Mutation (−), *n* = 30	Mutation (+), *n* = 14	*P*
CD4+ T cell count/μl	529 (276)	568 (597)	0.708	412 (415)	424 (648)	0.762
Log_10_ HIV-1 RNA, copies/ml	4.4 (2.7)	2.2 (1.9)	0.183	3.6 (2.7)	2.2 (1.7)	0.142

	Non-drug users					
	ART (−)			ART (+)		
	Mutation (–), *n* = 7	Mutation (+), n = 14	*P*	Mutation (−), *n* = 17	Mutation (+), *n* = 15	*P*
CD4+ T cell count/μl	989 (507)	455 (599)	*0.005*	351 (526)	554 (461)	0.350
Log_10_ HIV-1 RNA, copies/ml	3.6 (1.9)	4.8 (2.5)	0.313	4.3 (1.3)	4.3 (2.0)	0.852

Data presented are medians (IQR, interquartile range) in carriers and non-carriers of only minor protease resistance mutations. Statistical analysis was performed using Mann–Whitney test

Significant *P*-values are shown in italic

*ART (−)* anti-retroviral treatment-naive, *ART (+)* anti-retroviral treatment-experienced, *Mutation (+)* minor protease resistance mutation carrier, *Mutation (−)* minor protease resistance mutation non-carrier, *HIV-1* human immunodeficiency virus type

## Discussion

Effective use of protease inhibitors in HIV-1 treatment and prevention is largely impeded by the increasing development and spread of acquired and transmitted resistance [43]. Antiretroviral treatment-naive and -experienced IDUs are at an increased risk of acquiring antiretroviral drug resistance as a consequence of high risk sexual and injection practices [44]. In addition, increased selection of protease drug resistance in ART-experienced IDUs is linked to high drug injection intensity and poor adherence to treatment [35, 44]. Thus, continuous surveillance of drug resistance is important in planning for effective and successful treatment programs in IDUs. This study, determined PI resistance in ART-naive and -experienced IDUs in Mombasa City, in the coastal region in Kenya.

In the current study, major PI mutations were detected only in IDUs with the antiretroviral treatment-experienced (L90M, D30N and M46I) individuals having higher rates than -naive (D30N) individuals. Most importantly, major mutations, D30N and M46I co-existed with minor mutations, G48E, K20I and T74S, suggesting that these mutations mediate evolution of major drug resistance [45]. Our findings of L90M, D30N, and M46I mutations, are partly consistent with previous studies reporting on the presence of major PI mutations D30N and M46I in ART-naive sex workers, a most-at-risk-population from Nairobi, Kenya [34]. Our results are also, in part, consistent with previous studies in Canada showing D30N, M46I, and L90M PI resistance mutations among individuals reporting a history of injection drug use [46]. Likewise, our results are similar to previous studies showing presence of L90M, D30N, and M46I mutations among antiretroviral-naive IDUs in Rio de Janeiro, Brazil [47]. Importantly, phenotypic and/or clinical evidence indicate that D30N mutation confers high-level resistance to nelfinavir; L90M imparts resistance to nelfinavir, sequinavir, indinavir, and lopinavir; while M46I confers resistance to indinavir, lopinavir, fosamprenavir and nelfinavir [12, 39, 40]. In this study, most of the ART-experienced IDUs and non-drug users had been receiving first-line anti-retroviral treatment (NRTIs and NNRTIs) for at least a year, eliminating the possibility of cross-resistance to PIs [12]. Although not determined in the current study, it is possible that development of major PI resistance in ART-experienced IDUs is attributable to PI transmitted drug resistance, increased drug-injection frequency, particularly under the selection pressure of antiretroviral therapy [35]. Therefore, tipranavir/ritonavir and darunavir/ritonavir to which the above mutations have no effect [12] may be adopted as drugs of choice for second line treatment in Kenyan IDUs. Altogether, screening prior to initiating highly active antiretroviral therapy (HAART), monitoring of treatment and routine surveillance are important in achieving effective clinical care and preventing emergence and spread of resistance in this most-at-risk-population.

Overall, higher rates of minor mutations were found in IDUs relative to non-drug users, suggesting heterogeneity of HIV-1 strains in IDUs. The detection of A71T, G48E/R, I13V, K20I/R, L10I/V, L33F, L63P, T74S, V11I, and V32L minor mutations in this study is, in part, consistent with previous studies that reported K20R, L10I/V, L33F/I, and L63P minor mutations among HIV-positive women attending antenatal clinics in a large HIV treatment study in western Kenya [33]. In addition, our results corroborate previous studies showing K20I/M/R, L10I/V, I13V, and L63P minor mutations among HIV infected treatment-naive individuals in Morocco [48]. Of note are the higher rates of minor PI mutations in antiretroviral treatment-naive individuals in both the IDUs and non-drug users supporting a mechanism(s) of spontaneous emergence [49]. However, it is also possible that drug-related selective pressure enhances development of minor resistance in IDUs [35].

The high viral load among individuals carrying major PI mutations is consistent with phenotypic and clinical studies showing absence or low virologic response in patients having these major mutations [50]. It is important to emphasize that three of the individuals with major mutations concurrently harboured G48E, K20I, K20R and T74S minor mutations. This is not surprising because G48E inhibits as K20I, K20R and T74S enhance viral replication [12, 51]. In addition, CD4+ T cell counts were <500 cells/μl in three of the patients, indicating progression to immunodeficiency. Although minor PI mutations do not directly cause reduced phenotypic and/or clinical response to PI resistance [12], the lower CD4+ T cell counts observed in ART-naive users possibly suggests that minor mutations may contribute to viral fitness and infectivity in treatment-naive individuals [52]. This hypothesis is underscored by previous studies reporting that polymorphic compensatory mutations in the protease gene, may improve the replicative capacity of HIV-1 even in the absence of drug selective pressure or major resistance mutations [52]. Interestingly, previous studies have also reported low CD4+ T cell counts in anti-retroviral treatment naive IDUs harbouring minor PI mutations [53]. Therefore, our findings suggest that appearance of minor PI mutations may indicate disease progression among newly-infected individuals.

While a prospective design would have been important in examining the emergence of PI resistance in treatment-naive and -experienced IDUs, this cross-sectional study provides the first baseline evidence of occurrence of PI resistance in IDUs from Kenya. Although specific antiretroviral regimens used by the ART-experienced individuals were not evaluated in this study, poor adherence to ART by injection drug users, generate sub-optimal antiretroviral drug levels that exert selective pressure on the viral genome leading to resistance [44]. Since interactions of substances consumed and antiretroviral drugs in IDUs cause hepatic hyper-inflammation and derangements in metabolic functions [54, 55], toxicological analysis and monitoring of response to ART in a prospective approach will provide insights into the mechanisms underlying development of resistance. In addition, analysis of mutations outside the protease gene, such as *env* [56] that confer resistance to PI will identify additional mutants mediating resistance.

### Conclusion

Protease inhibitor transmitted drug resistance is likely to occur among Kenyan IDUs. Therefore, performing HIV-1 drug resistance analyses prior to initiating PI treatment in HIV patients from high-risk population groups is critical in achieving better treatment outcomes and prolonging the effectiveness of these drugs in the country.

## Sequence data

The 125 sequences described in this study were submitted to the GenBank under accession numbers: KP791995, KP791996, KP791997, KP791998, KP791999, KP792000, KP792001, KP792002, KP792003, KP792004, KP792005, KP792006, KP792007, KP792008, KP792009, KP792010, KP792011, KP792012, KP792013, KP792014, KP792015, KP792016, KP792017, KP792018, KP792019, KP792020, KP792021, KP792022, KP792023, KP792024, KP792025, KP792026, KP792027, KP792028, KP792029, KP792030, KP792031, KP792032, KP792033, KP792034, KP792035, KP792036, KP792037, KP792038, KP792039, KP792040, KP792041, KP792042, KP792043, KP792044, KP792045, KP792046, KP792047, KP792048, KP792049, KP792050, KP792051, KP792052, KP792053, KP792054, KP792055, KP792056, KP792057, KP792058, KP792059, KP792060, KP792061, KP792062, KP792063, KP792064, KP792065, KP792066, KP792067, KP792068, KP792069, KP792070, KP792071, KP792072, KP792073, KP792074, KP792075, KP792076, KP792077, KP792078, KP792079, KP792080, KP792081, KP792082, KP792083, KP792084, KP792085, KP792086, KP792087, KP792088, KP792089, KP792090, KP792091, KP792092, KP792093, KP792094, KP792095, KP792096, KP792097, KP792098, KP792099, KP792100, KP792101, KP792102, KP792103, KP792104, KP792105, KP792106, KP792107, KP792108, KP792109, KP792110, KP792111, KP792112, KP792113, KP792114, KP792115, KP792116, KP792117, KP792118, KP792119, KT033003, KT033004, KT033005, KT033006, KT033007, KT033008, and KT033009.
